# A retrospective analysis comparing persistence and adherence to treatment with free‐ vs fixed‐dose combination of an alpha blocker and an antimuscarinic agent in men with LUTS in Spain

**DOI:** 10.1111/ijcp.13616

**Published:** 2020-09-19

**Authors:** Margarita Landeira, Ana M. Mora Blázquez, Rodrigo Martins de Almeida, Patrick J. O. Covernton, José Medina‐Polo, Antonio Alcántara Montero

**Affiliations:** ^1^ Astellas Pharma S.A. Madrid Spain; ^2^ Astellas Pharma Europe Ltd. Chertsey, Addlestone Surrey UK; ^3^ Department of Urology Hospital Universitario Madrid Spain; ^4^ Manuel Encinas Health Center Cáceres Spain

## Abstract

**Introduction:**

Combination therapy with an alpha blocker (AB) plus an antimuscarinic (AM) is recommended for men with moderate‐to‐severe mixed lower urinary tract symptoms (LUTS) when monotherapy is not effective in relieving storage symptoms. This study compared treatment persistence and adherence with an AB plus AM fixed‐dose combination (FDC) vs an AB plus AM free‐dose combination in men with LUTS in Spain.

**Methods:**

Retrospective study using the Spanish IQVIA Cegedim Electronic Medical Records database. Men prescribed AB plus AM combination therapy were included in an FDC or free‐dose combination cohort based on their index treatment. Treatment persistence was the time from index date to first discontinuation of ≥1 of the two index drugs over 12 months. Adherence was measured using the fixed medication possession ratio (MPR).

**Results:**

Of 3114 patients identified, 999 were included (FDC, n = 790; free‐dose combination, n = 209). Median (95% CI) persistence was longer in the FDC (125 [109‐151] days) than in the free‐dose combination (31 [31‐36] days) cohort (hazard ratio [HR], 2.9; 95% CI, 2.4‐3.4; *P* < .0001). The 12‐month persistence rates were 31.1% (FDC cohort) and 8.9% (free‐dose cohort). The mean (SD) fixed MPR was higher in the FDC cohort (48.8 [37.2]) compared with the free‐dose cohort (23.1 [28.4]); more patients in the FDC cohort (34.2%) than in the free‐dose cohort (10.0%) were adherent (MPR ≥ 80%). The probability of treatment persistence and adherence increased with age (>80 vs <65 years, persistence HR, 0.7 [95% CI, 0.5‐0.9]; MPR difference, 12.5), polypharmacy (persistence HR, 0.7 [95% CI, 0.6‐0.9]; MPR difference, 10.7) and previous use of AB (persistence HR, 0.8 [95% CI, 0.7‐1.0]; MPR difference, 5.7) or AB/AM combinations (persistence HR, 0.7 [95% CI, 0.5‐0.9]; MPR difference, 11.1).

**Conclusions:**

Treatment with an AB/AM FDC is associated with better persistence and adherence vs a free‐dose combination in men with LUTS in Spain.

**What’s known**

- Combination therapy with an alpha blocker (AB) plus an antimuscarinic (AM) is recommended in men with lower urinary tract symptoms (LUTS) when monotherapy is not effective.
- Unfortunately, treatment persistence and adherence are low in patients receiving combination therapy.

**What’s new**

- Our data suggest that persistence and adherence increase in men with LUTS treated with a fixed‐dose combination vs a free‐dose combination of an AB plus an AM; this increase could improve patient outcomes and reduce healthcare costs.

## 1 INTRODUCTION

Lower urinary tract symptoms (LUTS) is a general term used to describe urinary symptoms originating from the bladder, prostate, urethra and/or adjacent pelvic floor or pelvic organs. LUTS may present as voiding symptoms (eg, hesitancy, straining and slow urinary stream), storage symptoms (eg, urgency, increased daytime frequency, nocturia, urgency urinary incontinence) and/or post‐voiding symptoms (eg, feeling of incomplete emptying, double‐voiding, post‐voiding urgency/incontinence).^1^, ^2^ Notably, approximately 50% of men experience “mixed” LUTS with both a voiding component (often attributed to benign prostatic obstruction [BPO] because of benign prostatic hyperplasia [BPH]) and a storage component (often attributed to overactive bladder [OAB]).^3^ The storage component is generally considered to be the most bothersome and can negatively impact patients’ physical and mental health, and their quality of life (QoL).^3^, ^4^, ^5^, ^6^

Available medications for the treatment of moderate‐to‐severe LUTS include alpha blockers (AB), antimuscarinic (AM) agents, 5‐phosphodiesterase inhibitors and 5‐alpha‐reductase inhibitors (5‐ARI). The European Association of Urology (EAU) guidelines recommend AB monotherapy (which primarily targets the voiding component) as first‐line pharmacological treatment for men with bothersome mixed LUTS.^7^ Furthermore, combination therapy with an AM plus an AB in men with moderate‐to‐severe LUTS is recommended when monotherapy with either drug is not effective in relieving storage symptoms.^7^ The increased efficacy of combination therapy vs monotherapy in improving storage symptoms has been demonstrated in clinical trials.^8^, ^9^, ^10^ Indeed, approximately two‐thirds of men with mixed LUTS require add‐on therapy with an OAB drug because of persistent bothersome symptoms, although in practice, the use of such combination therapy remains low.^11^, ^12^

Treatment persistence is a challenge for many chronic conditions and appears to be a particular issue with OAB drugs, especially AM agents.^13^, ^14^ Most patients receiving an AM agent will discontinue treatment within 1 year and median time to discontinuation is typically <5 months.^14^ In a study conducted in the UK, among patients ≥40 years with OAB receiving an AM, 1‐year persistence varied between 14% and 35%.^15^ Persistence with AB therapy appears to be better compared with AMs, but remains suboptimal, with typically <50% of patients remaining persistent at 1 year.^16^, ^17^ As poor persistence may be associated with worse outcomes and may lead to increased resource use, strategies to improve persistence in LUTS patients are an important area of research.^18^, ^19^

Treatment with fixed‐dose combination (FDC) tablets represents one means of improving persistence to multi‐drug therapy, including in men with mixed LUTS receiving an AB plus an AM. Tamsulosin 0.4 mg/solifenacin 6 mg FDC remains the only approved FDC of an AB (tamsulosin) plus an AM (solifenacin) available for the treatment of moderate‐to‐severe storage and voiding symptoms associated with BPH in men who are not adequately responding to treatment with monotherapy. A retrospective study conducted in the Netherlands showed that in patients with LUTS/BPH, the 12‐month persistence rate was significantly higher (51.3% vs 29.9%) among those taking an FDC of solifenacin/tamsulosin (Vesomni) vs a free‐dose combination.^20^ This FDC has been available in Spain since 2015, but no studies have been conducted to assess treatment persistence and adherence compared with free‐dose combination in a Spanish population. Using real‐world data, this study investigated the persistence and adherence to treatment with an FDC or a free‐dose combination of an AB plus an AM in men with LUTS in Spain.

## 2 METHODS

### 2.1 Study design and objectives

This was a retrospective, longitudinal, observational study conducted in Spain. Patients’ data were extracted from the Spanish IQVIA Cegedim Electronic Medical Records database. This national longitudinal database contains anonymised health records of patients collected through 8000 office‐based primary and secondary care physicians, covering approximately 3.0% of the Spanish population. Patient characteristics (age, gender, height and weight), diagnosis dates and codes, and prescriptions from primary and secondary care physicians were collected. Patients were classified into two cohorts (FDC and free‐dose combination) based on their index combination treatment with an AB and an AM. The index date was defined as the date of the first prescription of the FDC combination therapy, and for the free‐dose combination, if the AB and the AM drugs were not started on the same day but occurred within a 30‐day window, the index date would have been the date of the prescription of the second drug. Discontinuation was defined as no prescription renewal within a 30‐day grace period after the end of a prescription, and the date of discontinuation was defined as the end date of the last prescription. The primary objective of the study was to compare treatment persistence with an FDC of an AB plus an AM vs a free‐dose combination of AB plus AM over 12 months, with adjustment for patient characteristics at treatment initiation. Secondary objectives included comparing adherence between the two treatment groups and comparing treatment persistence and adherence with an FDC vs a free‐dose combination of tamsulosin/solifenacin.

### 2.2 Study population

Patients were men with LUTS aged ≥45 years at the index date who had received a combination therapy of an AB plus an AM, either as FDC or free‐dose combination from 1 February 2016 to 31 December 2016. Patients had to have ≥12 months of continuous enrolment before the index date without prescription of the same combination selected as index date combination therapy, and a 12‐month follow‐up period from the index date.

### 2.3 Endpoints

The primary endpoint was treatment persistence with an index FDC therapy of an AB plus an AM drug or a free‐dose combination of an AB plus an AM drug. Persistence was defined as the time from the index date to the first discontinuation of at least one of the two index drugs during a 12‐month follow‐up period. The persistence rate during the 12‐month follow‐up period was calculated as the proportion of patients who had not discontinued either of the two index drugs 12 months after the index date. Adherence to treatment with an FDC or with a free‐dose combination therapy was a secondary endpoint. Adherence over the 12‐month follow‐up period was measured using the fixed medication possession ratio (MPR)^21^ ([total number of days of supply of both index drugs during the post‐index period/2]/365 × 100). Patients were considered adherent if they had an MPR ≥ 80.0%.^22^ Adherence rate, defined as the proportion of adherent patients at the 12‐month post‐index date, was also calculated. Exploratory endpoints included (a) the proportion of men switching index combination therapy, where switching was defined as discontinuing one or both of the drugs of the index combination therapy and initiating a new drug of the same class within 30 days after discontinuation, and (b) assessment of healthcare resource utilisation during the 12‐month follow‐up period, including the number of general practitioner (GP) and specialist visits.

### 2.4 Statistical analysis

Demographic and baseline characteristics were summarised using descriptive statistics. Kaplan‐Meier analysis was used to describe the time to treatment discontinuation for the FDC and free‐dose combination groups. Discontinuation was the target event and censoring occurred in patients switching from an FDC to a free‐dose combination with the same molecules (ie, tamsulosin and solifenacin). Comparisons between FDC and free‐dose combinations for the time to discontinuation were conducted using univariate and multivariate Cox proportional hazards models adjusting for potential confounding factors. Hazard ratios (HRs) were estimated from these models using FDC as a reference, and represented the relative probability of stopping treatment. Univariate and multivariate analyses, using linear regression models, were used to compare the MPR between FDC and free‐dose combinations and investigate the impact of patient baseline characteristics. The patient characteristics to be included in the multivariate regression models were selected based on their clinical relevance and on the univariate analysis using a threshold level of *α* = 0.10. To compare the FDC vs free‐dose combinations, a stepwise approach was applied to identify the covariates to be used in the multivariate regression models. Sensitivity analyses were performed to test the impact of the definitions used in the study and were conducted by varying the grace period (considering treatment as discontinued with grace periods of 45, 60 and 90 days of the most recent prescription), the persistence definition, the combination definition and the prescriber's specialty.

## 3 RESULTS

### 3.1 Patient disposition

**FIGURE 1:**
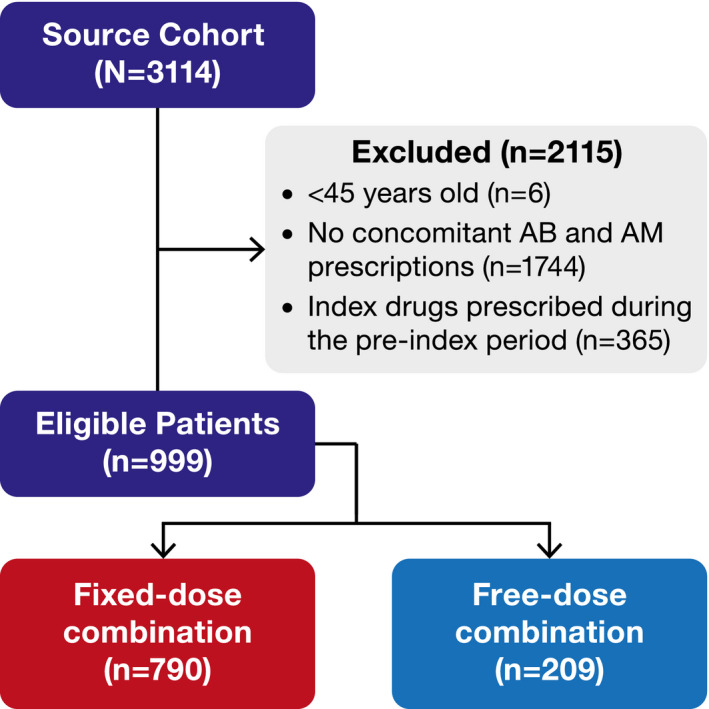
Patient Disposition. AB, alpha blocker; AM, antimuscarinic

Of 3114 patients identified in the source cohort, 999 patients were included (FDC, n = 790 [79.1%]; AB and AM free‐dose combination, n = 209 [20.9%]) and 2115 (67.9%) were excluded. Reasons for exclusion were patients aged <45 years (n = 6 [0.2%]), having no concomitant prescriptions of AB and AM during the study period (n = 1744 [56.0%]), and having index drugs prescribed concomitantly during the pre‐index period (n = 365 [11.7%]) (Figure 1).

### 3.2 Demographics and baseline characteristics

The mean (SD) age was lower among patients taking the FDC than among those taking the free‐dose combinations (69.4 [9.8] years vs 71.7 [9.5] years). The mean (SD) body mass index was comparable in the two groups (FDC, 30.3 [4.8]; free‐dose combination, 30.9 [4.5]). Urologists and GPs were the prescribers at the index date for 64.3% and 34.8% of patients, respectively, in the FDC group, and for 34.9% and 52.2%, respectively, in the free‐dose combination group. Compared with the FDC group, the free‐dose combination group had a higher rate of polypharmacy with ≥5 drugs (84.2% vs 70.9%) and a higher Charlson Comorbidity Index (Table 1).

**TABLE 1:** Demographics and baseline characteristics at the index date

Parameter	FDC (n = 790)	Free‐dose combination (n = 209)	Total (N = 999)	*P value*
Age, y				
Mean (SD)	69.4 (9.8)	71.7 (9.5)	69.9 (9.8)	.0024
Median	69.0	73.0	70.0	
Min, max	45.0, 94.0	46.0, 91.0	45.0, 94.0	
Age categories, n (%), y				.0036
<65	242 (30.6)	43 (20.6)	285 (28.5)	
65‐75	337 (42.7)	90 (43.1)	427 (42.7)	
>75	211 (26.7)	76 (36.4)	287 (28.7)	
BMI, kg/m^2^				.3131
n	222	66	288	
Mean (SD)	30.3 (4.8)	30.9 (4.5)	30.4 (4.7)	
Prescriber specialty				<.0001
General practitioner	275 (34.8)	109 (52.2)	384 (38.4)	
Urology	508 (64.3)	73 (34.9)	581 (58.2)	
Other	7 (0.9)	27 (12.9)	34 (3.4)	
Charlson Comorbidity Index, mean (SD)	1.2 (1.5)	1.9 (1.9)	1.4 (1.6)	<.0001
Polypharmacy, ATC number				.0015
0	31 (3.9)	1 (0.5)	32 (3.2)	
1	29 (3.7)	4 (1.9)	33 (3.3)	
2	43 (5.4)	3 (1.4)	46 (4.6)	
3	60 (7.6)	10 (4.8)	70 (7.0)	
4	67 (8.5)	15 (7.2)	82 (8.2)	
≥5	560 (70.9)	176 (84.2)	736 (73.7)	

Abbreviations: ATC, anatomical therapeutic chemical; BMI, body mass index; FDC, fixed‐dose combination; SD, standard deviation.

Overall, during the 12‐month pre‐index period, 51.5% (FDC, 47.7%; free‐dose combination, 65.6%) of patients had used ABs, 15.5% (FDC, 10.1%; free‐dose combination, 35.9%) used AMs, 2.0% (FDC, 1.3%; free‐dose combination, 4.8%) used 5‐ARIs and 7.8% (FDC, 5.9%; free‐dose combination, 14.8%) used combination therapies (Table 2).

**TABLE 2:** Treatment history during the 12‐month pre‐index period

Parameter	FDC (n = 790)	Free‐dose combination (n = 209)	Total (N = 999)
AB, n (%)	377 (47.7)	137 (65.6)	514 (51.5)
AM, n (%)	80 (10.1)	75 (35.9)	155 (15.5)
5‐ARI, n (%)	10 (1.3)	10 (4.8)	20 (2.0)
Combination therapy n (%)	47 (5.9)	31 (14.8)	78 (7.8)
Type of last combination n (%)^a^			
AB/5‐ARI	6 (12.8)	7 (22.6)	13 (16.7)
AB/AM	41 (87.2)	23 (74.2)	64 (82.1)
AB/AM/5‐ARI	0 (0.0)	1 (3.2)	1 (1.3)

Note

Percentages refer to the number of patients treated with each drug within each column.

Abbreviations: 5‐ARI, 5α‐reductase inhibitor; AB, alpha blocker; AM, antimuscarinic; FDC, fixed‐dose combination.

a Percentages refer to the number of patients receiving combination therapy.

The combination therapies prescribed at the index date are summarised in Table 3. In the free‐dose combination cohort, the most frequent combinations were tamsulosin plus solifenacin (n = 53, 25.4%) and tamsulosin plus oxybutynin (n = 49, 23.4%).

**TABLE 3:** Index drug combination prescribed at the index date

Parameter	FDC (n = 790)	Free‐dose combination (n = 209)	Total (N = 999)
Doxazosin/fesoterodine	0 (0.0)	10 (4.8)	10 (1.0)
Doxazosin/oxybutynin	0 (0.0)	12 (5.7)	12 (1.2)
Doxazosin/solifenacin	0 (0.0)	17 (8.1)	17 (1.7)
Silodosin/solifenacin	0 (0.0)	16 (7.7)	16 (1.6)
Tamsulosin/fesoterodine	0 (0.0)	18 (8.6)	18 (1.8)
Tamsulosin/oxybutynin	0 (0.0)	49 (23.4)	49 (4.9)
Tamsulosin/solifenacin	790 (100.0)	53 (25.4)	843 (84.4)
Others	0 (0.0)	34 (16.3)	34 (3.4)

Note

Data are reported as n (%).

Abbreviation: FDC, fixed‐dose combination.

### 3.3 Treatment persistence

**FIGURE 2:**
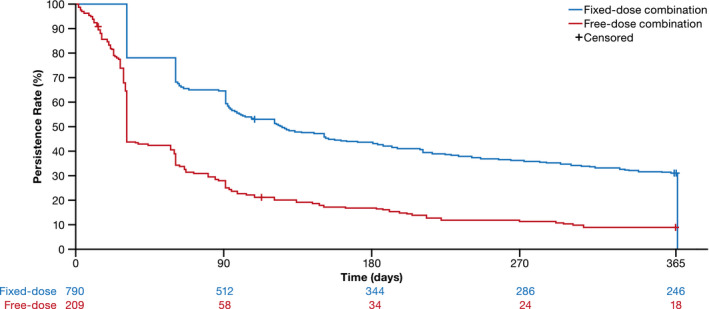
Kaplan‐Meier Curve of Time to Treatment Discontinuation With FDC and Free‐Dose AB Plus AM Combination Prescribed at the Index Date. AB, alpha blocker; AM, antimuscarinic; FDC, fixed‐dose combination

Based on Kaplan‐Meier analysis, the median (95% CI) time to discontinuation was 125 [109‐151] days in the FDC cohort and 31 [31‐36] days in the free‐dose combination cohort. The highest rate of discontinuation was observed during the first 30 days, particularly in the free‐dose combination cohort (Figure 2).

The 12‐month persistence rate was 31.1% in the FDC cohort and 8.9% in the free‐dose cohort. Multivariate Cox regression analysis showed that patients in the FDC cohort were approximately three times less likely to discontinue combination treatment than those in the free‐dose combination cohort (HR, 2.9; 95% CI, 2.4‐3.4; *P* < .0001). The probability of treatment persistence increased with age; patients aged >80 years were more likely to remain on treatment than those <65 years (HR, 0.7; 95% CI, 0.5‐0.9; *P* = .0023). The probability of remaining on treatment also increased with the presence of ≥5 drugs in the treatment history (HR, 0.7; 95% CI, 0.6‐0.9; *P* = .0003), and the previous use of an AB (HR, 0.8; 95% CI, 0.7‐1.0; *P* = .0314) and combinations of an AB plus an AM (other than the index therapy) or an AB plus a 5‐ARI (HR, 0.7; 95% CI, 0.5‐0.9; *P* = .0227) (Table 4).

**TABLE 4:** Univariate and multivariate Cox regression models to compare probability of treatment discontinuation between FDC and free‐dose AB plus AM drug combinations

	Univariate Cox		Multivariate Cox	
	HR (95% CI)	*P* value	HR (95% CI)	*P* value
Cohort				
FDC	Ref			
Free‐dose combination	2.6 (2.2‐3.0)	<.0001	2.9 (2.4‐3.4)	<.0001
Age categories, years				
<65	Ref			
65‐80	0.8 (0.7‐1.0)	.0373	0.9 (0.7‐1.0)	.0932
>80	0.7 (0.5‐0.9)	.0012	0.7 (0.5‐0.9)	.0023
Prescriber specialty				
General practitioner	Ref			
Urology	0.8 (0.6‐0.9)	.0024		
Other	1.6 (1.1‐2.3)	.0132		
Specialty				
Primary care	Ref			
Secondary care	0.8 (0.7‐0.9)	.0097		
Polypharmacy (≥5 drugs)				
No	Ref			
Yes	0.8 (0.6‐0.9)	.0013	0.7 (0.6‐0.9)	.0003
Previous AB				
No	Ref			
Yes	0.9 (0.7‐1.0)	.0407	0.8 (0.7‐1.0)	.0314
Previous combination				
No	Ref			
Yes	0.8 (0.6‐1.0)	.068	0.7 (0.5‐0.9)	.0227

Abbreviations: AB, alpha blocker; AM, antimuscarinic; FDC, fixed‐dose combination; HR, hazard ratio; Ref, reference.

The results of all sensitivity analyses confirmed that patients on FDC treatment were significantly less likely to discontinue compared with those treated with a free‐dose combination, with HRs between 2.0 and 3.2 in the multivariate analyses (*P* < .0001).

### 3.4 Treatment adherence

The mean (SD) fixed MPR was higher in the FDC cohort (48.8 [37.2]) compared with the free‐dose cohort (23.1 [28.4]), and more patients in the FDC cohort were adherent (MPR ≥ 80%; n = 270/790, 34.2%) compared with the free‐dose cohort (n = 21/209, 10.0%). After adjustment for potential confounders such as patient age, specialty, polypharmacy and previous treatment, the multivariate linear model confirmed that patients in the free‐dose cohort had a significantly lower MPR than those in the FDC cohort (difference [95% CI], −30.1 [−35.5 to −24.7]; *P* < .0001). Patients aged 65‐80 years (difference, 6.5; *P* = .0117) or >80 years (difference, 12.5; *P* = .0004) vs <65 years, patients having ≥5 drugs in their medical history (difference, 10.7; *P* < .0001), and those having previous treatment with an AB (difference, 5.7; *P* = .0132) or with a combination therapy of an AB plus an AM (other than the index therapy), or an AB plus a 5‐ARI (difference, 11.1; *P* = .0089) were more adherent to their index treatments (Table 5).

**TABLE 5:** Univariate and multivariate linear regression models to compare adherence of FDC and free‐dose AB plus AM drug combination

	Univariate Linear Model		Multivariate Linear Model	
	Coeff (95% CI)	*P* value	Coeff^a^ (95% CI)	*P* value
Cohort				
FDC	Ref			
Free‐dose combination	−25.7 (−31.1 to −20.3)	<.0001	−30.1 (−35.5 to −24.7)	<.0001
Age categories, years				
<65	Ref			
65‐80	6.8 (1.5‐12.1)	.0113	6.5 (1.4‐11.5)	.0117
>80	13.1 (5.9‐20.2)	.0003	12.5 (5.6‐19.4)	.0004
Prescriber specialty				
General practitioner	Ref			
Urology	6.6 (1.9‐11.4)	.0061		
Others	−12.8 (−25.7 to 0.1)	.0526		
Specialty				
Primary	Ref			
Secondary	5.6 (0.9‐10.3)	.0205		
Polypharmacy (≥5 drugs)				
No	Ref			
Yes	11.0 (5.8‐16.2)	<.0001	10.7 (5.6‐15.8)	<.0001
AB previous				
No	Ref			
Yes	5.1 (0.5‐9.7)	.0307	5.7 (1.2‐10.2)	.0132
Use of combination therapy^b^				
No	Ref			
Yes	9.9 (1.3‐18.5)	.0233	11.1 (2.8‐19.4)	.0089

Abbreviations: 5‐ARI, 5α‐reductase inhibitor; AB, alpha blocker; AM, antimuscarinic; CI, confidence interval; Coeff, coefficient; FDC, fixed‐dose combination; Ref, reference.

a The differences in multivariate linear models are adjusted by patient age, specialty, polypharmacy and previous treatment (AB/combination therapy).

b The use of combinations includes AB plus AM (other than the index therapy) and AB plus 5‐ARI.

All sensitivity analyses performed using different values of the grace period, combination definitions and prescriber's specialty confirmed that patients treated with FDC had a significantly higher average MPR compared with those treated with free‐dose combinations. The coefficient estimates associated with the free‐dose cohort in the linear model ranged from −31.6 to −19.5 (*P* < .0001) in the multivariate analysis.

### 3.5 Persistence and adherence to treatment with solifenacin plus tamsulosin

Kaplan‐Meier analysis to assess the 12‐month treatment persistence for patients receiving an FDC tablet vs a free‐dose combination of solifenacin plus tamsulosin showed a longer median (95% CI) time to discontinuation in the FDC cohort (125 [109‐151] days) (Figure 2) than in the free‐dose combination cohort (31 [31‐46] days). Similarly, the persistence rate was higher in the FDC cohort (31.1%) than in the solifenacin plus tamsulosin free‐dose combination cohort (12.7%). The multivariate Cox proportional hazards model demonstrated that patients in the FDC cohort were approximately three times less likely to discontinue treatment than those in the free‐dose combination cohort (HR, 2.7; 95% CI, 1.9‐3.6; *P* < .0001). The probability of treatment persistence was higher in patients aged 65‐80 years (HR, 0.8; 95% CI, 0.7‐1.0; *P* < .05) or >80 years (HR, 0.6; 95% CI, 0.5‐0.8; *P* < .0012) vs those aged <65 years, in those having ≥5 drugs in the treatment history (HR, 0.7; 95% CI, 0.6‐0.9; *P* = .0011), and in those with previous use of an AB (HR, 0.8; 95% CI, 0.7‐0.9; *P* = .0077) and combination therapy of an AB plus an AM (other than the index therapy) or an AB plus a 5‐ARI (HR, 0.6; 95% CI, 0.4‐0.9; *P* = .0102) (Table 6).

**TABLE 6:** Univariate and multivariate Cox regression models to compare probability of treatment discontinuation between FDC and free‐dose combinations of solifenacin plus tamsulosin

	Univariate Cox		Multivariate Cox	
	HR (95% CI)	*P* value	HR (95% CI)	*P* value
Cohort				
FDC	Ref			
Free‐dose combination	2.5 (1.8‐3.4)	<.0001	2.7 (1.9‐3.6)	<.0001
Age categories, years				
<65	Ref			
65‐80	0.8 (0.6‐0.9)	.0024	0.8 (0.7‐1.0)	.0481
>80	0.6 (0.4‐0.7)	<.0001	0.6 (0.5‐0.8)	.0012
Prescriber specialty				
General practitioner	Ref			
Urology	0.8 (0.7‐1.0)	.015		
Other	2.2 (1.2‐4.2)	.0138		
Specialty				
Primary care	Ref			
Secondary care	0.8 (0.7‐1.0)	.0257		
Polypharmacy (≥5 drugs)				
No	Ref			
Yes	0.7 (0.6‐0.8)	<.0001	0.7 (0.6‐0.9)	.0011
Previous AB				
No	Ref			
Yes	0.8 (0.6‐0.9)	.0008	0.8 (0.7‐0.9)	.0077
Previous combination				
No	Ref			
Yes	0.5 (0.4‐0.8)	.0026	0.6 (0.4‐0.9)	.0102

Abbreviations: AB, alpha blocker; FDC, fixed‐dose combination; HR, hazard ratio; Ref, reference.

Similarly, after adjusting for potential confounders, a multivariate analysis using linear models confirmed that patients in the free‐dose cohort had a significantly lower MPR than did those in the FDC cohort (difference, −31.3; *P* < .0001). Treatment adherence was higher for patients aged 65‐80 years (difference, 7.2; *P* = .0112) or >80 years (difference, 14.2; *P* = .0003) compared with those aged <65 years, patients with ≥5 drugs in the treatment history (difference, 10.2; *P* = .0004), and in those previously treated with AB (difference, 9.0; *P* = .0003) or AM (difference, 12.2; *P* = .0020).

### 3.6 Treatment switching

Of the 734 (FDC, 545; free‐dose combination, 189) patients who discontinued their index treatment during the post‐index period, 209 (28.5%) switched to a different therapy of the same class (this excludes patients who stopped FDC and started tamsulosin and/or solifenacin as monotherapy or free‐dose combination, or free‐dose tamsulosin/solifenacin patients who started FDC). The proportion of patients who switched their index treatment was higher in the free‐dose cohort (n = 148/189, 78.3%) than in the FDC cohort (n = 61/545, 11.2%). Overall, 11 (5.7%), 151 (72.2%) and 47 (22.5%) patients who discontinued switched to a new AB plus AM combination, a new AB in monotherapy, or a new AM in monotherapy, respectively.

### 3.7 Healthcare resource utilisation

Most patients (99.5%) in both study cohorts had ≥1 GP visit. The proportion of patients who had ≥1 urologist visit was 57.2% in the FDC cohort and 45.5% in the free‐dose combination cohort, and the mean (SD) number of visits was similar in both cohorts (FDC, 1.5 [2.2]; free‐dose combination, 1.6 [3.3]; *P* = .0825). The proportion of patients who had ≥1 visit with other specialists was 47.8% and 59.8% in the FDC and free‐dose combination cohorts, respectively.

## 4 DISCUSSION

Despite the negative impact of LUTS on patients’ QoL and the evidence that available treatments are effective, many patients with mixed storage and voiding symptoms may not be receiving optimal care.^3^, ^4^, ^5^, ^6^, ^8^, ^9^, ^10^ Firstly, the use of add‐on therapy with an OAB drug (eg, an AM) to target unresolved storage symptoms in patients treated with an AB remains low and, secondly, even in those patients receiving appropriate combination treatment, drug adherence and persistence can be poor.^11^, ^12^, ^13^, ^14^ This retrospective study investigated the persistence and adherence to pharmacological treatment with an AB plus an AM, and compared the use of an FDC vs a free‐dose combination. The FDC cohort showed a longer median (95% CI) time to treatment discontinuation (125 [109‐151] days vs 31 [31‐36] days) and a higher 12‐month persistence rate (31.1% vs 8.9%) compared with the free‐dose combination cohort. The highest discontinuation rate occurred during the first 30 days and was higher in the free‐dose cohort than in the FDC cohort. Since this study was based on database records on prescriptions, it is important to note that, in the FDC cohort, both drugs were prescribed as a single tablet at the index date with a 30‐day prescription; therefore, no discontinuation could be recorded for the first 30 days. On the contrary, most of the patients (>90%) in the free‐dose cohort were prescribed the first of the two drugs before the index date, and this drug could have been discontinued at any time within the first 30 days after the index date. The short grace period used for withdrawal and the use of two tablets in the free‐dose combination vs a single tablet in the FDC cohort may also have contributed to the difference observed between cohorts. Similarly, adherence (mean [SD] MPR, 48.8 [37.2] vs 23.1[28.4]) and adherence rate (MPR ≥ 80%, 34.2% vs 10.0%) were higher in the FDC cohort than in the free‐dose combination cohort, respectively. Similar results were found when comparing patients treated with an FDC vs a free‐dose combination of solifenacin and tamsulosin. Furthermore, the probability of treatment persistence and adherence increased with age, history of polypharmacy and previous use of an AB and combination therapy (AB/AM and AB/5‐ARI). These findings did not change when the data were analysed using variations in the duration of the grace period, the definitions of combination therapy and the prescriber's specialty. Approximately two‐thirds of patients with mixed symptoms have been reported to require add‐on therapy to manage storage symptoms after 4 weeks of AB monotherapy.^12^ The efficacy of combination therapy with an AB plus an AM in reducing symptoms and improving health‐related QoL in men with LUTS has been demonstrated in multiple clinical trials.^9^, ^10^, ^23^ A real‐world study of men with LUTS/BPH not responding to monotherapy showed that once‐daily treatment with an FDC of solifenacin 6 mg plus tamsulosin 0.4 mg resulted in clinically meaningful improvement in OAB symptoms bother and health‐related QoL, as measured by OAB‐q (OAB questionnaire), and was well tolerated.^24^

Our findings are in line with those from a similar retrospective, observational cohort of men with LUTS/BPH treated with an AB plus an AM in the Netherlands that showed a longer time to first discontinuation with an FDC vs a free‐dose combination (414 days vs 112 days; HR, 2.04; 95% CI, 1.77‐2.35) with a higher 12‐month persistence rate (51.3% vs 29.9%).^20^ However, although absolute levels of persistence and persistence rates reported in that study were higher compared with our results, we obtained a greater relative difference between FDC and free‐dose combination therapy (HR of 2 vs 3, respectively). This may be because of differences in the respective healthcare systems or the databases used to derive the data in the two studies. Taken together, these findings suggest that prescribing an FDC treatment with a single tablet in place of a combination of two tablets may be a way to help patients with mixed storage and voiding LUTS remain on treatment longer. In line with this, the advantage of using an FDC vs a free‐dose combination has been demonstrated for other pathologies. A meta‐analysis study of data published between 2000 and 2017 comparing FDC vs free‐dose combination treatment for hypertension showed that patients treated with an FDC had a higher treatment persistence (HR, 1.84 [95% CI, 1.00‐3.39]) and adherence (mean difference, 14.92% [95% CI, 7.38%‐22.46%]) to their hypertension medication compared with those treated with a free‐dose combination.^25^

There are differences in the management of LUTS/BPH between urologists and GPs, which may be because of disparities in training and knowledge of the most recent clinical trials and current guideline recommendations, as well as access to diagnostic resources. Urologists are generally more likely to prescribe medical therapy for LUTS than GPs (64.5% vs 47.1%), as suggested by an observational study of the BPH Registry and Patient Survey conducted in the United States.^26^ In the current study, a greater proportion of patients in the FDC cohort were treated by urologists vs GPs. However, sensitivity analyses showed that the persistence advantage of FDC over free‐dose combination treatment was maintained irrespective of the prescriber specialty, suggesting that FDC therapy for mixed LUTS can play a useful role in primary care. Nevertheless, as fewer patients treated by GPs vs urologists may receive adequate diagnostic evaluation and therapy, there remains a need for better training to recognise LUTS symptoms and educational programs for GPs to effectively treat patients with LUTS.^26^, ^27^, ^28^ In this regard, a program developed in Spain that included the establishment of diagnostic protocols and referral practices, as well as continuous training measures and cooperation between GPs and urologists with periodic evaluation of the outcomes and measures, resulted in positive outcomes with a reduction in the number of referrals for LUTS and improved referral adequacy from 46% to 65%.^29^

The cost associated with LUTS management is considerable in Europe, and a study conducted in Spain reported that healthcare consumption increases with symptom severity, suggesting that better symptom control may result in lower healthcare costs.^30^ Two studies conducted in Spain and in the UK demonstrated the cost‐effectiveness of an FDC of solifenacin 6 mg plus tamsulosin 0.4 mg compared with a free‐dose combination of tolterodine plus tamsulosin in patients with moderate‐to‐severe LUTS/BPH. In both studies, the use of solifenacin plus tamsulosin FDC was associated with lower annual total treatment costs per patient than the use of a free‐dose combination of tolterodine plus tamsulosin (£860 vs £959 and € 1,349 vs € 1,619, respectively).^31^, ^32^

Some limitations need to be considered when evaluating the findings of this study. Patients in the FDC cohort received only tamsulosin and solifenacin, whereas those in the free‐dose combination cohort were treated with different drug combinations, with 23.4% receiving oxybutynin. It is possible that the less tolerable side effects associated with oxybutynin may have contributed to the lower persistence and adherence observed in the free‐dose combination cohort vs the FDC cohort. However, the secondary analysis comparing FDC tamsulosin‐solifenacin with free‐dose tamsulosin‐solifenacin alone provides similar findings to the overall analysis, although the study was not powered to specifically investigate secondary endpoints and the solifenacin doses were different for the FDC (6 mg) and free‐dose combination (5 mg or 10 mg). The retrospective study design may introduce limitations because patient selection biases can be introduced. Since this was not a randomised study, there were differences in some of the baseline characteristics between the FDC and the free‐dose combination cohorts that might have influenced the outcome. In particular, the proportion of patients who seemed not to be taking any treatment at the index date was higher in the FDC group than in the free‐dose combination group (Table 2), although patients may have been on therapy prior to the 12 months before index date. Also, since this is a database study, certain information including disease severity, concomitant medications and reasons for discontinuation may not have been easily accessible. Importantly, data on persistence and adherence were based on the dates of prescription and the number of pills per pack, and no monitoring was performed regarding whether patients were following the therapy as prescribed, or even if it was collected at the pharmacy. A diagnosis of LUTS/BPH/OAB was not part of the inclusion criteria in this study; therefore, it is possible that doxazosin and terazosin could have been prescribed for hypertension rather than LUTS in some patients. Furthermore, the small sample size of this study should be considered when evaluating the effect of age, polypharmacy and previous use of an AB or AB/AM/5‐ARI combination. The reasons for the higher switch rate to different AB and/or AM molecules upon discontinuation in the free‐dose cohort vs the FDC cohort remain unclear and further study on subsequent LUTS management in the post‐discontinuation period among patients who did and did not switch is warranted.

Overall, this study showed that index treatment persistence and persistence rate, as well as average MPR and adherence rate, are higher among patients treated with an FDC vs a free‐dose AB plus AM combination. The probability of treatment persistence and adherence increases with age, polypharmacy with ≥5 drugs in the treatment history, and the previous use of an AB or AB/AM or 5‐ARI combination, and the proportion of patients switching one or both agents was higher among those treated with a free‐dose combination vs an FDC. These findings, along with those from other studies, suggest that optimising the prescription of AB/AM combination therapy through the use of an FDC—possibly by raising awareness among GPs to recognise patients with unresolved storage symptoms and appreciate that treatment with an FDC may improve persistence—may result in better outcomes for patients and in the optimisation of healthcare resource utilisation.

Even though further research is necessary to better understand the potential benefits of FDC vs free‐dose combination therapy to treat LUTS/BPH, this study provides valuable information to help physicians in Spain and other countries where FDC AB/AM treatment is available in decision‐making. The improved persistence provided by FDC AB/AM treatment has the potential to improve patient outcomes and reduce healthcare costs.

## DISCLOSURES

A. Alcántara Montero reports personal fees from Astellas Pharma, Inc. outside the submitted work. R. Martins de Almeida, A.M. Mora Blázquez, P.J.O. Covernton and M. Landeira are employed by Astellas Pharma, Inc. J. Medina‐Polo has nothing to disclose.

## AUTHOR CONTRIBUTIONS

M. Landeira and P.J.O. Covernton contributed to the conception and design of the study. M. Landeira conducted the data acquisition. A.M. Mora Blázquez, J. Medina‐Polo, M. Landeira and P.J.O. Covernton contributed to the analysis and interpretation of the data. A. Alcántara Montero, A.M. Mora Blázquez, M. Landeira, P.J.O. Covernton and R. Martins de Almeida contributed to the drafting of the article. All authors provided critical revisions and final approval.

## Data Availability

Researchers may request access to anonymised participant level data, trial level data and protocols from Astellas sponsored clinical trials at www.clinicalstudydatarequest.com. For the Astellas criteria on data sharing see: https://clinicalstudydatarequest.com/Study‐Sponsors/Study‐Sponsors‐Astellas.aspx.
